# Ethanol ablation of cystic thyroid nodules: institutional experience with one year follow-up

**DOI:** 10.1007/s11547-026-02216-x

**Published:** 2026-05-06

**Authors:** Lorenzo Scappaticcio, Nicole Di Martino, Pamela Ferrazzano, Miriam Longo, Luca Marinelli, Giovanni Docimo, Maria Ida Maiorino, Giuseppe Bellastella, Salvatore Cappabianca, Katherine Esposito

**Affiliations:** 1 Department of Advanced Medical and Surgical Sciences, University of Campania “Luigi Vanvitelli”, 80138 Naples, Italy; 2 Unit of Endocrinology and Metabolic Diseases, AOU University of Campania “Luigi Vanvitelli”, Naples, Italy; 3 Department of Life Science, Health, and Health Professions, Link Campus University, 00165 Rome, Italy; 4 Department of Precision Medicine, University of Campania ‘Luigi Vanvitelli’, Naples, Italy; 5 Unit of Thyroid Surgery, AOU University of Campania “Luigi Vanvitelli”, Naples, Italy

**Keywords:** Thyroid nodule, Ethanol ablation, Efficacy outcomes

## Abstract

**Background:**

We aimed to identify the main factors influencing the achievement of high efficacy or negative outcomes for ethanol ablation (EA) of cystic thyroid nodules.

**Methods:**

We conducted a retrospective cohort study on consecutive patients treated with EA for cytologically benign and symptomatic CTNs at the Vanvitelli University Hospital in Naples (Italy) over a period of four years. Data was analyzed using multivariable logistic regression and Fisher's exact test. All tests were performed at the significant level of 0.05.

**Results:**

We included 118 nodules undergoing EA with one year follow-up [median volume 16.2 (7.0–32.8) mL], with important cervical symptomatology [overall visual analogue scale (VAS) score 26.0 (22.0–30.0) and cosmetic score (CS) 4.0 (3.0–4.0)]. At 12 months follow-up (T12), median VRR was 88.9 (77.2–97.6) %, overall VAS score and CS were 0 (0.0–1.5) and 1.0 (1.0–1.0), respectively. The classical EA was the only predictor of VRR > 75% (OR = 3.27; 95% CI 1.01–10.57; *p* = 0.048). Monolocular aspect was the only predictor (OR = 5.719; 95% CI 1.664–19.652; *p* = 0.006) of VAS = 0 and CS = 1.

**Conclusions:**

EA for CTNs is usually associated with high efficacy and rarely (one out of ten patients) with negative outcomes at one-year follow-up. The classical EA procedure (when the cystic content can be initially aspirated) and the monolocular aspect thyroid nodules seem to be factors that positively influence the high efficacy of EA, regardless of baseline nodule volume, ultrasound composition, number of EA sessions, and total ethanol amount.

**Supplementary Information:**

The online version contains supplementary material available at 10.1007/s11547-026-02216-x.

## Introduction

Alternative treatments to surgery and, in selected cases, the first-line therapies for benign, locally symptomatic thyroid nodules (TNs) are represented by ultrasound (US)-guided minimally invasive treatments (MITs), as ethanol ablation (EA) and other thermal ablation techniques [1–3]. EA is regarded as the treatment of choice for relapsing, symptomatic, benign purely cystic thyroid nodules (CTNs; i.e., cystic component > 90%) or for predominantly cystic thyroid nodules (PCTNs; i.e., cystic portion > 50%) [1–3]. EA provides a substantial volume reduction ratio (VRR), which typically ranges from 80 to 100% in purely CTNs and from 65 to 85.4% in PCTNs [4, 5], with the benefits being sustained over time [4, 5]. A substantial body of research has assessed the efficacy of EA for CTNs, primarily focusing on VRR and, to a lesser extent, on the amelioration of local symptomatology [5]. Moreover, EA can be recognized as a safe technique, with complications being transient and well manageable. Yet, we have recently demonstrated that EA of symptomatic and benign CTNs lead to a great improvement of health-related quality of life (HRQoL) [6].

Standardization of EA procedure for cystic thyroid nodules remains a challenge, as several major technical issues are yet to be resolved. For instance, the optimal technique for managing cystic thyroid nodules with highly viscous content, that is difficult to aspirate, remains to be defined. In order to facilitate liquid aspiration, larger or open-window intervention needles could be employed, with or without repeated irrigation with room-temperature saline [7–11]; alternatively, the injection of ethanol prior to aspiration may be performed to reduce the viscosity of CTNs [12–16] (a phenomenon called by someone the Saint Januarius effect, referring to the miracle of the liquefaction of the blood of St. Januarius, bishop, martyr, and patron saint of Naples, Italy) (https://www.catholicnewsagency.com). Moreover, the optimal starting dose of ethanol for each session of EA to treat CTNs is still debated [15, 17], and it is not known if similar ethanol doses may result in equal VRR and therapeutic success ratio (TSR) [1–3, 17]. In clinical practice, the effectiveness of EA in shrinking thyroid nodules appears to be influenced by several factors, including the nodule composition (CTNs vs. PCTNs), the baseline nodule volume, the aspirated liquid volume, the retention or the aspiration of ethanol after evacuating the internal cystic content, the nodule vascularity, the nodule aspect (i.e., presence of septations in multilocular nodules), and the duration of follow-up [1–3, 17]. 

Therefore, the purposes of this retrospective cohort study on EA for CTNs with one year follow-up were: (1) to evaluate the VRR, the TSR, the local symptom improvement, the regrowth percentage, the final approach (i.e., US follow-up or surgery); (2) to identify the main factors influencing the achievement of high efficacy or negative outcomes.

## Methods

### Patients with benign and symptomatic CTNs undergoing EA

This is an observational study that consecutively involved patients who underwent EA for cytologically benign and symptomatic CTNs. The study was conducted at the endocrine outpatient clinics of the University Hospital of Campania “Luigi Vanvitelli” in Naples (Italy) from June 1, 2021, to October 31, 2025. We included men and women aged ≥ 18 years, who were euthyroid at baseline, had negative serum calcitonin, and were planning to undergo EA with the aim of reducing thyroid nodule volume. In our institution, EA was performed as first-line therapy for symptomatic CTNs that recurred after one aspiration attempt, while surgery was recommended when EA failed (defined as early recurrence, persistence of high volume, or relevant local symptomatology, after multiple EA sessions). For PCTNs, benignity was established via cytological analysis of the solid component.

Exclusion criteria were (1) pregnancy; (2) lack of complete follow-up (defined as less than 12 months of follow-up); and (3) denial of informed consent to participate in the study. Anthropometric and sociodemographic parameters, and details on EA complications, were obtained by chart review. Thyroid nodule volume was assessed by ultrasound and calculated using the half-cube formula, as recommended by ATA guidelines [18].

EA was performed by the same experienced operator (L.S., with 7 years of experience) using a 23-gauge needle and consisted of one to two punctures for each session. The procedure was performed under ultrasound guidance using a lateral approach, without the administration of local anaesthesia. Anticoagulant therapy was suspended for a period of ten days prior to EA, while antiplatelet agents were maintained in their current dosage. The internal fluid of CTNs was aspirated as much as possible by using a syringe of 10 mL or 20 mL or 50 mL. Ninety-six percent ethanol was injected before [contrariwise EA (contrEA)] or after cystic content aspiration [classical EA (classEA)] in the amount of 5–30% of the initial nodule volume (≤ 10 mL for each session), without early ethanol reaspiration. The exact volume of ethanol injected was decided during the procedure, based on the following features: the aspirated liquid volume, the nodule location, the thickness of the cystic wall, and the patient tolerance. Total ethanol amount corresponded to the ethanol used to treat a CTN in one or more EA sessions. ContrEA was performed when the aspiration of liquid content was initially not possible after two attempts, one with a needle of 23 Gauge and the second with a needle of 22 Gauge. ContrEA involved that ethanol was left inside the nodule for one week, which consequently allowed the successful aspiration (Saint Januarius effect). After the successful aspiration of the cystic content, no additional doses of ethanol were injected. The absence of ethanol reinjection is a deliberate protocol choice, primarily aimed at reducing complications. Patients were discharged after a 30-min observation period.

The patient follow-up, consisting of clinical assessment and thyroid nodule ultrasound, was provided four time during the first year (after one, three, six and twelve months from the EA procedure), and then once a year, as indicated by consensus statement [1]. Any repetitions of EA procedure were performed after two or three months from the first treatment to additionally reduce the liquid content or when the nodules regrown. Regrowth was defined as a more than 50% increase in total volume compared to the previously reported smallest volume on US. The current prospective study was approved by the Institutional Review Board of University of Campania “Luigi Vanvitelli” (Naples, Italy) (Campania 2, 2024 0039/i).

### Outcomes

The VRR was obtained by dividing the reduction in nodule volume (baseline minus follow-up) by the baseline volume and multiplying the result by 100%. TSR was define as achieving a volume loss greater than 50% at the 12-month follow-up. The severity of local symptoms was evaluated using a Visual Analogue Scale (VAS), with patients rating their experience of neck pain, dysphagia, sensation of a foreign body, general discomfort, and cough on a scale ranging from 0 (absent) to 10 (severe) [1]. The physician evaluated the Cosmetic Score (CS) using a four-point scale: [1] 1, absence of palpable nodule; 2, palpable nodule without cosmetic concern; 3, a cosmetic issue occurring only during swallowing; and 4, easily observable cosmetic alteration. In the current study, we retrieved the VRR, the symptom scores and cosmetic scores at baseline and one-year follow-up visit. Because EA is known to be typically associated with high VRRs, we arbitrarily set the high efficacy at 75% in terms of VRR. The high efficacy outcomes for each nodule one year after EA were the following: VRR > 75%, VAS overall score of 0 and cosmetic score equal to 1; when at least one of these last three outcomes was reached, EA was associated with high local efficacy. The final approach was the last management option adopted for the patient, defined as the US follow-up or the surgery. Negative outcomes were the following: non-achievement of TSR after one year, regrowth within one year, and final approach corresponding to surgery.

### Statistical analysis

Descriptive statistics were used to analyse the demographic and clinical characteristics of all participants in the study sample. The Kolmogorov–Smirnov test was used to assess the distribution of variables. Continuous variables were presented as mean and standard deviation (SD) or median and interquartile ranges (IQR) according to sample distribution. Categorical variables were presented as frequencies and percentages. The primary analyses aimed to identify predictors for the three main efficacy outcomes 12 months after EA. For each of these three outcomes, a multivariable logistic regression model was developed. Primary model was built to evaluate the main predictors of interest. This model included baseline nodule volume (mL), ultrasound composition (purely cystic vs predominantly cystic), ultrasound aspect (monolocular vs multilocular), and type of EA procedure (classical vs contrariwise procedure). To adjust for potential confounding factors, each primary model included the following covariates: number of EA sessions, and total ethanol amount (mL). Results were reported as odds ratios (OR) with 95% confidence intervals (CI). Analyses for negative outcomes were also carried out: here, multivariable logistic regression was found to be statistically inappropriate due to the low number of observed events (n < 10), which resulted in complete or quasi-complete separation in the data [19]. Therefore, these outcomes were analysed at a descriptive (prevalence) and univariate level using Fisher's exact test.

A p-value lower than 0.05 was considered statistically significant. All statistical analyses were performed using SPSS software (version 27.0, SPSS, Chicago, IL, USA).

## Results

### Institutional cohort

**Fig. 1:**
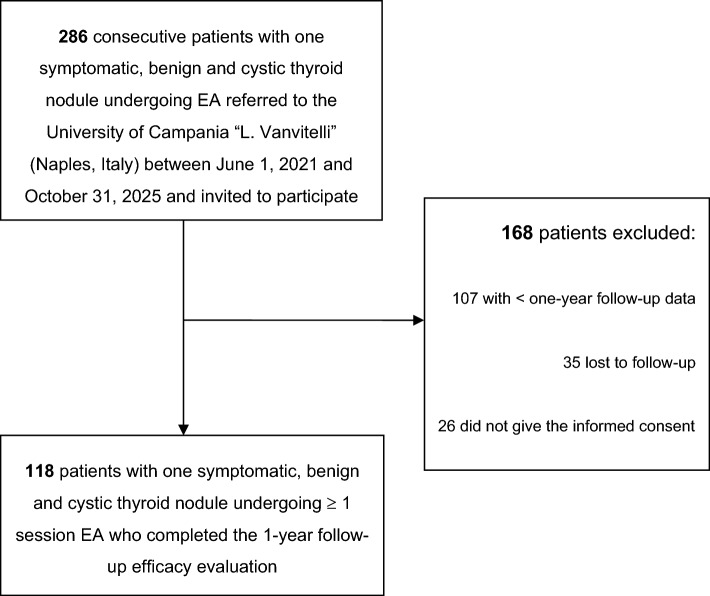
Flow diagram for recruitment and follow-up of patients diagnosed with symptomatic, benign and cystic thyroid nodules, and receiving EA for the purpose of volume reduction

As shown in Fig. 1, between June 1, 2021, to October 31, 2025, from 286 consecutive patients with one symptomatic, benign cystic thyroid nodule undergoing EA, we excluded 168 patients.

We finally included 118 nodules undergoing EA with one year follow-up [median volume 16.2 (7.0–32.8) mL], from 118 patients [71 females (60.2%) and 47 males, median age 49.5 (42.5–60.3) years] with cervical symptomatology [overall VAS score 26.0 (22.0–30.0) and CS 4.0 (3.0–4.0)]. Regarding ultrasound characteristics, 69 participants (58.5%) had purely CTNs, and 95 (80.5%) nodules had a monolocular aspect. ClassEA procedure was used in 97 participants (82.2%). At 6 months follow-up (T6), the median nodule volume was 2.4 (1.2–8.0) mL, with median VRR of 81.4 (62.3–92.8)%. At this timepoint, 104 participants (88.1%) achieved TSR, and median overall VAS score and CS were 5.0 (0.0–7.0) and 1.0 (1.0–2.0), respectively. At 12 months follow-up (T12), the median nodule volume was 1.0 (0.3–2.9) mL, with median VRR of 88.9 (77.2–97.6)%. At this timepoint, 107 participants (90.7%) achieved TSR, and median overall VAS score and CS were 0 (0.0–1.5) and 1.0 (1.0–1.0), respectively. Minor complications occurred in 27 participants (22.9%), with a low incidence of major complications (0.85% in the study cohort). Table 1 summarizes the demographic, clinical, and technical characteristics of the study population.

**Table 1:** Clinical, demographic and technical characteristics of the included patients undergoing ethanol ablation (EA) for benign and purely cystic thyroid nodules (CTNs) or predominantly cystic thyroid nodules (PCTNs)

Variables	Study cohort (n = 118)
Age, years	49.5 (42.5–60.3)
*Gender, n (%)*	
Female	71 (60.2)
Male	47 (39.8)
BMI, Kg/m^2^	24.5 (22.0–27.5)
*Ultrasound composition of nodules, n (%)*	
Purely cystic	69 (58.5)
Predominantly cystic	49 (41.5)
*Ultrasound appearance of nodules, n (%)*	
Monolocular aspect	95 (80.5)
Multilocular aspect	23 (19.5)
*Type of EA procedure, n (%)*	
Classical procedure	97 (82.2)
Contrariwise procedure	21 (17.8)
Aspirated volume, ml	13.0 (4.0–23.0)
Total ethanol amount, mL	2.2 (1.2–3.7)
*Sessions of EA, n*	1.0 (1.0–1.0)
Single session, n (%)	89 (75.4)
2 EA sessions, n (%)	19 (16.1)
3 EA sessions, n (%)	9 (7.6)
4 EA sessions, n (%)	1 (0.9)
*Baseline Nodule size*	
Volume, mL	16.2 (7.0–32.8)
Dmax, mm	39.6 ± 13.0
*Nodule size, after 6 months*	
Volume, mL	2.4 (1.2–8.0)
VRR, %	81.4 (62.3–92.8)
*Nodule size, after 12 months*	
Volume, mL	1.0 (0.3–2.9)
VRR, %	88.9 (77.2–97.6)
Number of nodules achieving TSR	
T6	104 (88.1)
T12	107 (90.7)
*VAS, overall score*	
VAS score T0	26.0 (22.0–30.0)
VAS score T6	5.0 (0.0–7.0)
VAS score T12	0 (0.0–1.5)
*Cosmetic score*	
Cosmetic score T0	4.0 (3.0–4.0)
Cosmetic score T6	1.0 (1.0–2.0)
Cosmetic score T12	1.0 (1.0–1.0)
*Final outcome, n (%)*	
*US follow-up*	114 (96.6)
*Surgery*	4 (3.4)
*Complications, n (%)*	
Minor Complications	27 (22.9)
Major complications	1 (0.85)

Continuous variables are expressed in median and interquartile range (IQR), and categorical variables with numbers and percentages

BMI, body mass index; Dmax, maximum diameter; VRR, volume reduction ratio; TSR, therapeutic success ratio (VRR > 50%); VAS, visual analogue scale; T0, baseline (before ea); T6, 6 months after EA; T12, 12 months after EA; US, ultrasound

Minor complications: 16 cases of transient burning sense, 7 cases of local pain, 2 cases of neck hematoma, 2 cases of set of symptoms including dizziness/drunkenness and/or flushing and/or increased heartbeat and/or nausea and/or headache. Major complications: 1 case of transient dysphonia

### *VRR* > *75%*

Table 2 depicts the multiple logistic regression model for predictors of achieving VRR > 75%. In the primary model, type of EA procedure (classEA, OR = 3.87; 95% CI 1.32-11.37; *p* = 0.014) and baseline nodule volume (OR = 0.986; 95% CI 0.975–0.997; *p* = 0.016) were found to be significant predictors. Following adjustment for number of EA sessions and total ethanol amount, the classEA remained the only independent significant predictor of success (OR = 3.27; 95% CI 1.01–10.57; *p* = 0.048) (Supplementary table S1).

**Table 2:** Multiple logistic regression model for predictors of achieving volume reduction ratio (VRR) > 75% in the study cohort

VRR	β coefficient	OR	95% CI	*p*-value
US composition of nodules (= purely cystic)	0.266	1.305	0.506; 3.365	0.582
US aspect of nodules (= monolocular)	0.950	2.585	0.873; 7.652	0.086
Thyroid nodule volume, mL	-0.014	0.986	0.975; 0.997	0.016
Type of EA procedure (= classical)	1.353	3.868	1.316; 11.366	0.014

CI, Confidence interval; OR, odds ratio; US, ultrasound; EA, Ethanol ablation

US composition of nodules: 0 predominantly, 1 purely cystic (predominantly as reference category). US aspect of nodules: 0 multilocular, 1 monolocular (multilocular as reference category). Type of EA procedure: 0 contrariwise, 1 classical (contrariwise as reference category)

### *Overall VAS score* = *0*

Table 3 shows the multiple logistic regression model used to identify predictors of achieving overall VAS of 0. The analysis identified the monolocular aspect (OR = 6.322; 95% CI 2.228–17.939; *p* < 0.001) and the baseline nodule volume (OR = 0.983; 95% CI 0.971–0.995; *p* = 0.005) as significant predictors. After adjusting for number of EA sessions and total ethanol amount, monolocular aspect resulted as the only significant independent predictor (OR = 5.719; 95% CI 1.664–19.652; *p* = 0.006) (Supplementary table S2).

**Table 3:** Multiple logistic regression model for predictors of achieving overall visual analogue scale (VAS) score = 0 in the study cohort

VAS score	β coefficient	OR	95% CI	*p*-value
US composition of nodules (= purely cystic)	0.356	1.428	0.586; 3.477	0.433
US aspect of nodules (= monolocular)	1.844	6.322	2.228; 17.939	< 0.001
Thyroid nodule volume, mL	−0.018	0.983	0.971; 0.995	0.005
Type of EA procedure (= classical)	0.863	2.370	0.779; 7.214	0.129

CI, Confidence interval. OR, odds ratio. US, ultrasound; US, ultrasound; EA, Ethanol ablation

US composition of nodules: 0 predominantly, 1 purely cystic (predominantly as reference category). US aspect of nodules: 0 multilocular, 1 monolocular (multilocular as reference category). Type of EA procedure: 0 contrariwise, 1 classical (contrariwise as reference category)

### *CS* = *1*

In the multiple logistic regression model, monolocular aspect (OR = 9.005; 95% CI 2.756–29.424; *p* < 0.001) and baseline nodule volume (OR = 0.980; 95% CI 0.967–0.992; *p* = 0.002) emerged as significant predictors of achieving CS of 1 (Table 4). In the adjusted model, which included sessions of EA and total ethanol amount, monolocular aspect (OR = 3.919; 95% CI 1.029–14.922; *p* = 0.045) was the only significant independent predictor (Supplementary table S3).

**Table 4:** Multiple logistic regression model for predictors of achieving cosmetic score (CS) = 1 in the study cohort

Cosmetic score	β coefficient	OR	95% CI	*P* value
US composition of nodules (= purely cystic)	0.485	1.624	0.538; 4.900	0.390
US aspect of nodules (= monolocular)	2.198	9.005	2.756; 29.424	< 0.001
Thyroid nodule volume, mL	−0.021	0.980	0.967; 0.992	0.002
Type of EA procedure (= classical)	0.720	2.054	0.517; 8.162	0.306

CI, Confidence interval; OR, odds ratio; US, ultrasound; EA, Ethanol ablation

US composition of nodules: 0 predominantly, 1 purely cystic (predominantly as reference category). US aspect of nodules: 0 multilocular, 1 monolocular (multilocular as reference category). Type of EA procedure: 0 contrariwise, 1 classical (contrariwise as reference category)

### Negative outcomes

**Fig. 2:**
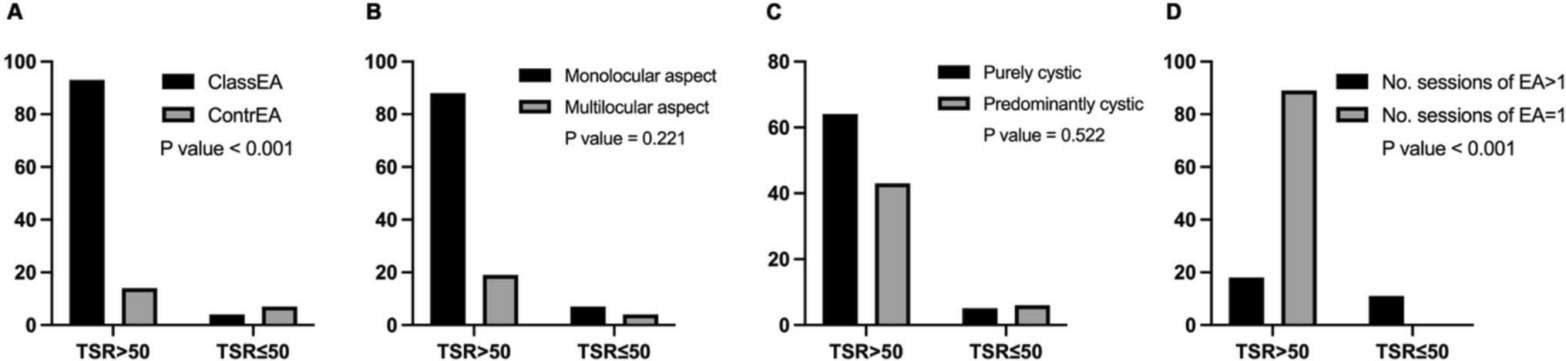
Fisher’s exact test between therapeutic success ratio (TSR) > 50% and type of ethanol ablation (EA) procedure (**A**) [classical EA (classEA) and contrariwise EA (contrEA)], aspect (**B**) and composition (**C**) of cystic thyroid nodules at ultrasound, and number of EA sessions (**D**) in the study cohort

Twelve months after EA, eleven participants (9.3%) did not achieve TSR, and four participants (3.4%) required surgery. There were no cases of nodule regrowth during the one-year follow-up (Table 1). Figure 2A shows a significant association between TSR ≤ 50% and type of EA procedure (*p* < 0.001). Specifically, the treatment failure (TSR ≤ 50%) occurred in 33.3% (7/21) of cases treated with the ContrEA procedure, compared to 4.1% (4/97) of cases treated with ClassEA procedure. No significant association was observed between TSR ≤ 50% and aspect of thyroid nodules at US (*p* = 0.221). Although the failure rate was numerically higher in multilocular nodules (17.4% [4/23]) than in monolocular nodules (7.4% [7/95]), this difference was not statistically significant (Fig. 2B). Similarly, Fig. 2C shows that there was no significant association between TSR ≤ 50% and composition of CTNs at US (p = 0.522).

Figure 2D shows a significant association (*p* < 0.001) between the number of EA sessions and the TSR ≤ 50%.

**Fig.3:**
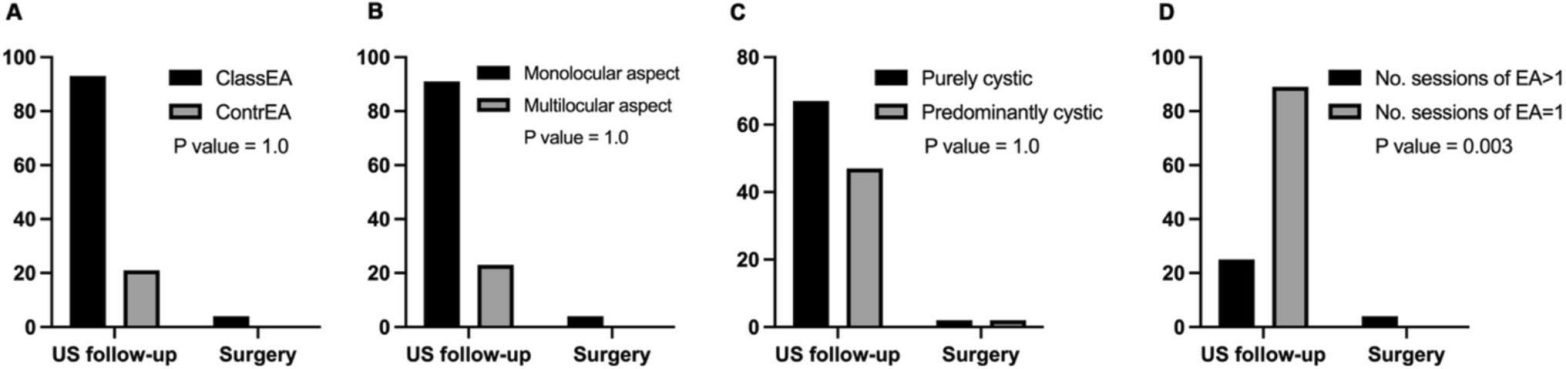
Fisher’s exact test between final outcome [ultrasound (US) follow-up or surgery] and type of ethanol ablation (EA) procedure (**A**) [classical EA (classEA) and contrariwise EA (contrEA)], aspect (**B**) and composition (**C**) of cystic thyroid nodules at ultrasound, and number of EA sessions (**D**) in the study cohort

No significant association was found between surgery and type of EA procedure (*p* = 1.0), aspect (*p* = 1.0), or composition (*p* = 1.0) of thyroid nodules at US (Fig. 3A, 3B, 3C). Figure 3D shows a significant association between surgery and the number of EA sessions (*p* = 0.003).

## Discussion

The international standardization of EA techniques for benign and symptomatic CTNs is needed; [1–3, 17]. To enable this goal, several crucial technical issues should be addressed, including the definition of optimal EA strategies and the ethanol amount in cases when aspiration is not possible [1–3, 12, 13, 17]. Moreover, there is a paucity of evidence regarding factors that exert a significant influence on the outcomes (favorable or unfavorable outcomes) of EA in clinical practice [1–3, 17].

Over a period of four years, we reported our clinical experience with the use of EA in the management of benign and symptomatic CTNs, with a follow-up period of one year. Specifically, we retrospectively analyzed our study cohort (n = 118 CTNs) to assess the high efficacy and negative outcomes of EA procedures and their influencing factors.

First, consistent with the current literature [1–5], we found that EA showed excellent results both in terms of VRR and improvement of local symptomatology: starting from large (median maximum diameter 39 mm and volume 16.2 mL) and very symptomatic (median overall VAS score 26 and CS 4) CTNs, we obtained a VRR of more than 80% after six months, which further increased to almost 90% after 12 months; similarly, median overall VAS score and CS became 10 and 1, after six months, respectively, with disappearance of symptomatology after 12 months, as expressed by median overall VAS score 0 and CS 1. Instead regarding the failure of EA in shrinking CTNs, this occurred in about 12% of cases: TSR was not reached 12 months after EA in 9% of participants, and surgery was undertaken in 3% of cases because of early recurrence (within two months of the ethanol injection) after multiple EA sessions. However, true regrowth did not occur in all cases which initially responded to EA treatment. Complications of EA were typically minor, transient and well manageable, in accordance with what we recently demonstrated by a dedicated study to the safety of EA [20].

The real novelty of the current study consisted in exploring potential factors [i.e., baseline nodule volume, ultrasound composition (purely cystic vs predominantly cystic), ultrasound aspect (monolocular vs multilocular), type of EA procedure (classical vs contrariwise procedure), number of EA sessions, and total ethanol amount] impacting on the high efficacy (VRR > 75%, overall VAS score = 0, CS = 1) and negative (non-achievement of TSR after one year, regrowth within one year, and final approach corresponding to surgery) outcomes of EA. This issue is poorly and heterogeneously explored in literature [16, 17, 21–24]. In this respect, we demonstrated that the classEA procedure was the only independent and significant predictor of achieving VRR > 75%. In other words, compared to the contrEA procedure and the other factors, the classEA procedure was associated with a higher (about three times more) probability to achieve therapeutic success in terms of VRR > 75%. One main explanation of this last finding could be that in the contrEA procedure no additional ethanol doses were injected after the aspiration of the cystic content. In other studies [12–14, 16], when the cystic content was impossible to aspirate or it was just minimally aspirated, additional doses of ethanol were injected inside the nodule after aspiration: here, the EA procedure was named “two-stage EA” since a repeat EA was performed 1–3 months after the initial ethanol injection for the liquefaction of viscous contents [16]. And as for both overall VAS score = 0 and CS = 1, we found that the US aspect of CTNs, and particularly the monolocular aspect, was the only true predictor of disappearance of cervical symptomatology, compared to the multilocular one (about four-six times more). We acknowledge that the presence of internal septations, dividing the cystic nodule into multiple compartments, could hamper the spread of ethanol and its necrotic effect, which ultimately weakens the EA procedure. In this respect, we could assume that some ways to improve the efficacy of EA for multilocular CTNs could include a greater competence of operators in disrupting the cyst septations and the use of higher ethanol amount.

Moreover, although the negative outcomes were rare (about one out of ten patients), we attempted to identify the main factors associated with EA failure. Specifically, we found that multiple EA sessions were associated with both the non-achievement of TSR after one year, and final approach corresponding to surgery. In clinical practice, the interpretation of this finding could correspond to the evidence that CTNs non-responding to the ablative treatment are typically submitted to multiple EA sessions. In fact, the four patients (3% of our study cohort) early switching to surgery underwent four, three, and two EA sessions in one, two and one cases, respectively. However, it should be emphasized that these four CTNs were very large, with a median volume of 171.5 (160.5–184) mL and maximum diameter of 85.5 (82.0–90.5) mm. So, in this regard, although we had too few negative outcome data for statistical purposes, we could assume that the very large size of CTNs could negatively matter in the efficacy of EA. Finally, we found that the contrEA procedure was significantly associated with TSR ≤ 50%: this could be always explained by the fact that the aspirated content also included ethanol and no further ethanol doses were injected, compared to “two-stage EA” [16]. These findings could represent an incremental contribution to current clinical practice and procedural recommendations for EA of CTNs.

Our study does have some limitations. First, we did not explore the nodule vascularity as potential factor influencing the efficacy of EA [1–3, 22]. Second, follow-up time could be considered short (one year) [22], precluding conclusions on long-term durability beyond the observation period. Third, our findings refer to the aspiration of cystic content executed with 22–23 Gauge needles. We think that thin needles can be effective devices for aspiration of CTNs and they are associated with negligible pain for patients when local anesthesia is not adopted. This also aligns with EA regarded as a MIT that is easy to perform (i.e., no local anesthesia, thin needles). However, the superiority of larger over thinner needles in aspiration of CTNs has never been proved. Fourth, we did not consider the non-achievement of the disappearance of cervical symptomatology as a negative outcome: this is because, compared to VRR, it could suffer from subjectivity of both patients and doctors and comorbidities of patients. Supporting this, it is well known that the improvement of local symptomatology depends on the increase of VRR, and not vice versa.

## Conclusions

Based on the results of this study, EA for CTNs is usually associated with high efficacy and rarely (one out of ten patients) with negative outcomes at the one-year follow-up. The classical EA procedure (when the cystic content can be initially aspirated) and the monolocular aspect thyroid nodules seem to be factors that positively influence the high efficacy of EA, regardless of baseline nodule volume, ultrasound composition, number of EA sessions, and total ethanol amount.

## Supplementary Information

Below is the link to the electronic supplementary material.

Supplementary file1 (DOCX 27 kb)

## Data Availability

The datasets used and/or analyzed during the current study are available from the corresponding author upon reasonable request.
