# Supplementary material for: Ethanol ablation of cystic thyroid nodules: institutional experience with one year follow-up
Source: Radiol Med. 2026 May 6;131(7):1228–36. doi: 10.1007/s11547-026-02216-x (PMC13369035; doi:10.1007/s11547-026-02216-x)
Supplement: Supplementary file 1 — Supplementary file1 (DOCX 27 kb) [file 11547_2026_2216_MOESM1_ESM.docx]

**Ethanol ablation of cystic thyroid nodules: institutional experience with one year follow-up**

**Supplementary Table S1**. Multiple logistic regression model for predictors of achieving volume reduction ratio (VRR) > 75%, after adjusting for gender, number of sessions of EA, and total ethanol amount in the study cohort

| **VRR** | **β coefficient** | **OR** | **95% CI** | **p-value** |
| --- | --- | --- | --- | --- |
| US composition of nodules (=purely cystic) | 0.276 | 1.318 | 0.398; 4.367 | 0.652 |
| US aspect of nodules (=monolocular) | 0.665 | 1.944 | 0.484; 7.803 | 0.349 |
| Thyroid nodule volume, mL | 0.006 | 1.006 | 0.976; 1.037 | 0.707 |
| Type of EA procedure (=classical) | 1.183 | 3.265 | 1.009; 10.568 | 0.048 |
| Sessions of EA | -0.751 | 0.472 | 0.162; 1.377 | 0.169 |
| Total ethanol amount, mL | -0.094 | 0.910 | 0.720; 1.149 | 0.429 |

CI, Confidence interval. OR, odds ratio; US, ultrasound; EA, Ethanol ablation.

Type of EA procedure: 0 contrariwise, 1 classical (contrariwise as reference category), US composition of nodules: 0 predominantly, 1 cystic (predominantly as reference category). US aspect of nodules: 0 multilocular, 1 monolocular (multilocular as reference category).

**Supplementary Table S2**. Multiple logistic regression model for predictors of achieving visual analogue scale (VAS) score = 0, after adjusting for gender, session of ethanol ablation, and total ethanol amount in the study cohort

| **VAS score** | **β coefficient** | **OR** | **95% CI** | **p-value** |
| --- | --- | --- | --- | --- |
| US composition of nodules (=purely cystic) | 0.362 | 1.437 | 0.483; 4.271 | 0.514 |
| US aspect of nodules (=monolocular) | 1.744 | 5.719 | 1.664; 19.652 | 0.006 |
| Thyroid nodule volume, mL | -0.011 | 0.989 | 0.964; 1.015 | 0.389 |
| Type of EA procedure (=classical) | 0.793 | 2.210 | 0.685; 7.130 | 0.185 |
| Sessions of EA | -0.170 | 0.843 | 0.301; 2.366 | 0.746 |
| Total ethanol amount, mL | -0.040 | 0.960 | 0.777; 1.187 | 0.708 |

CI, Confidence interval; OR, odds ratio; US, ultrasound; EA, Ethanol ablation.

Type of EA procedure: 0 contrariwise, 1 classical (contrariwise as reference category), US composition of nodules: 0 predominantly, 1 cystic (predominantly as reference category). US aspect of nodules: 0 multilocular, 1 monolocular (multilocular as reference category).

**Supplementary Table S3**. Multiple logistic regression model for predictors of achieving cosmetic score (CS) = 1, after adjusting for gender, session of ethanol ablation, and total ethanol amount in the study cohort

| **Cosmetic score** | **β coefficient** | **OR** | **95% CI** | **P value** |
| --- | --- | --- | --- | --- |
| US composition of nodules (=purely cystic) | -0.095 | 0.909 | 0.240; 3.438 | 0.888 |
| US aspect of nodules (=monolocular) | 1.366 | 3.919 | 1.029; 14.922 | 0.045 |
| Thyroid nodule volume, mL | -0.029 | 0.971 | 0.933; 1.010 | 0.147 |
| Type of EA procedure (=classical) | 0.387 | 1.473 | 0.337; 6.433 | 0.607 |
| Sessions of EA | -0.329 | 0.719 | 0.224; 2.315 | 0.581 |
| Total ethanol amount, mL | -0.016 | 0.984 | 0.736; 1.316 | 0.912 |

CI, Confidence interval. OR, odds ratio; US, ultrasound; EA, Ethanol ablation.

Type of EA procedure: 0 contrariwise, 1 classical (contrariwise as reference category), US composition of nodules: 0 predominantly, 1 cystic (predominantly as reference category). US aspect of nodules: 0 multilocular, 1 monolocular (multilocular as reference category).
